# Auditory Processing Abilities of Parkinson's Disease Patients

**DOI:** 10.1155/2017/2618587

**Published:** 2017-05-04

**Authors:** Robert L. Folmer, Jay J. Vachhani, Sarah M. Theodoroff, Rachel Ellinger, Amy Riggins

**Affiliations:** ^1^National Center for Rehabilitative Auditory Research, VA Portland Medical Center, Portland, OR, USA; ^2^Department of Otolaryngology, Oregon Health & Science University, Portland, OR, USA; ^3^Department of Communication Sciences & Disorders, Northwestern University, Evanston, IL, USA; ^4^Department of Communication Sciences & Disorders, University of Wisconsin, Madison, WI, USA

## Abstract

Since Parkinson's Disease (PD) primarily affects older people, a majority of PD patients have age-related hearing loss (HL) that will worsen over time. The goal of this study was to assess peripheral and central auditory functions in a population of PD patients and compare the results with a group of age-matched control subjects. Study participants included 35 adults with PD (mean age = 66.9 ± 11.2 years) and a group of 35 healthy control subjects (mean age = 65.4 ± 12.3 years). Assessments included questionnaires, neuropsychological tests, audiometric testing, and a battery of central auditory processing tests. Both study groups exhibited patterns of sensorineural hearing loss (slightly worse in the PD group) which were typical for their age and would contribute to difficulties in communication for many participants. Compared to the control group, PD patients reported greater difficulty in hearing words people are speaking. Although 27 PD patients (77%) were good candidates for amplification, only 7 (26%) of these hearing aid candidates used the devices. Because it is important for PD patients to optimize communication with their family members, caregivers, friends, and clinicians, it is vital to identify and remediate auditory dysfunction in this population as early as possible.

## 1. Introduction

Parkinson's Disease (PD) affects approximately 1 million people in the United States and more than 10 million people worldwide [1]. The average age for PD onset is approximately 60 years, and the prevalence of PD increases with age [2]. Since the majority of people who are 60 years old or older have significant hearing loss (HL) and the prevalence of HL increases with age [3, 4], a majority of PD patients have significant HL that will worsen over time.

Cognitive decline and dysfunction are common sequelae of PD [2]. Untreated hearing loss is also associated with poorer cognitive functioning and can contribute to dementia [5–10]. For patients with untreated hearing loss, more of their resources are dedicated to auditory perceptual processing to the detriment of other cognitive processes such as working memory. Hearing loss may contribute to dementia through exhaustion of cognitive reserves, social isolation, sensory deafferentation, or a combination of these mechanisms [11]. Therefore, untreated hearing loss is likely to exacerbate cognitive dysfunction that is experienced by many PD patients.

Ziemssen and Reichmann [12] stated that “although non-motor symptoms such as sensory dysfunctions are also common and disabling manifestations of the disease, they are often not formally assessed and thus are frequently misdiagnosed or under diagnosed.” It is likely that auditory deficits are often not diagnosed or addressed in PD patients because hearing tests are either not conducted or considered during patient evaluations. Evaluations of central auditory processing are not likely to be administered at all. In their review of sensory symptoms in PD, Santos-García et al. [13] recommended that “hearing dysfunction must be considered in patients with Parkinson Disease.”

Previous studies by Yýlmaz et al. [14], Vitale et al. [15], Lai et al. [16], and Pisani et al. [17] reported greater hearing loss in PD patients compared to control groups without PD. Also, Guehl et al. [18], Lewald et al. [19], and Vitale et al. [20] reported impaired auditory processing in PD patients compared to control groups.

The goal of this study was to assess peripheral and central auditory functions in a population of PD patients and compare the results with a group of age-matched control subjects without PD or other neurological disorders. Unlike previous investigations involving PD patients, this study implemented a comprehensive test battery to assess auditory processing. The current study also conducted neuropsychological assessments to correlate with auditory and demographic data.

## 2. Methods

All procedures for recruitment, informed consent, and conduct of the study adhered to the requirements of the Institutional Review Board at VA Portland Medical Center where the study was conducted between 2012 and 2015.

Participants included 35 adults who had a medical diagnosis of PD and 35 age-matched control subjects who did not have PD or any other neurological disorders. PD patients were recruited from clinics at VA Portland Medical Center and Oregon Health & Science University (OHSU). After written informed consent was obtained, participants underwent the procedures and assessments described below over the course of three separate sessions.

### 2.1. Questionnaires

All participants completed the following questionnaires.

*Hearing History Questionnaire* [21] is a self-administered instrument in which participants reported any history or complaints of hearing loss/auditory dysfunction. This questionnaire also captured information related to hearing aid use, auditory disease, or surgery.

*Hearing Handicap Inventory for Adults* (HHIA, [22]) is a 25-item questionnaire that assesses the emotional and social consequences of auditory dysfunction.

### 2.2. Assessments of PD Severity

The Hoehn and Yahr [23] and Schwab and England [24] scales were used to assess the stage and severity of PD for individuals in the patient group.

PD patients were also asked to rate their abilities “during the past week” for 12 activities such as swallowing, handwriting, dressing, hygiene, falling, salivating, turning in bed, walking, and cutting food (these questions were taken from Part II of the Unified Parkinson's Disease Rating Scale [25]).

### 2.3. Neuropsychological Tests

*Wide Range Achievement Test 4* (WRAT-Reading, Psychological Assessment Resources, Inc., Lutz, FL) measures word decoding through word recognition. This test is an estimator of intelligence and also measures learning ability/disability. The WRAT was included to obtain an estimate of general intelligence, which is useful for interpretation of other neuropsychological and auditory assessments.

*Rey Auditory Verbal Learning Test *(RAVLT, [26]) evaluates a variety of functions: short-term auditory verbal memory, rate of learning, learning strategies, retroactive and proactive interference, presence of confabulation or confusion in memory processes, retention of information, and differences between learning and retrieval. Participants are given a list of 15 unrelated words repeated over five different trials and are asked to repeat them. Another list of 15 unrelated words is given and the subject must then repeat the original list of 15 words; this process is repeated again 30 minutes later.

*Beck Depression Inventory II* (BDI-II, [27]) is a 21-item self-administered questionnaire that assesses the presence and severity of depression.

### 2.4. Comprehensive Audiometric Evaluation

The ear canals and tympanic membranes of each participant were visually inspected with an otoscope. Pure tone air and bone conduction thresholds were measured in each ear using the American Speech-Language-Hearing Association-recommended procedure [28].

### 2.5. Assessments of Central Auditory Processing

*Speech intelligibility in noise *was assessed using the Words in Noise (WIN) test [29] in which 2 lists of 25 words are presented to each ear in the presence of background masking noise. Participants repeated each word they heard.

Computerized versions of the following tests were administered in which the audio tracks from CDs were triggered by a program written in Matlab (MathWorks, Natick, MA) and played through a digital-to-analog converter and amplifier connected to the inputs of a GSI-61 clinical audiometer (Grason-Stadler, Eden Prairie, MN). The sounds were then delivered to the listener via ER3A insert earphones (Etymotic Research, Inc., Elk Grove Village, IL). Playing, pausing, and repeating of test items were controlled from a computer screen interface. Tests were conducted at a minimum of 35 dB sensation level (i.e., 35 dB above the threshold level at which speech is detectable).

Responses were indicated by the participant using either a verbal response or computer touchscreen tap. Participant responses were immediately entered by the tester using a graphical version of the appropriate score sheet that was displayed on a computer screen. Scoring and storage of results were performed by the computer program, as was the randomization of the order in which the tests were conducted. Subjects were encouraged to take breaks, and testing was discontinued if fatigue or frustration was evident. This portion of the testing protocol lasted approximately two hours.

*Staggered-Spondaic-Word (SSW) Test [30]*. Each SSW item is made up of two spondaic words, presented in a way that creates four test conditions: (1) right noncompeting (RNC), (2) right competing (RC), (3) left competing (LC), and (4) left non-competing (LNC). Therefore, the SSW evaluates dichotic listening, word integration, and separation abilities. Participants repeat as many of the four words (or two spondaic words) as possible.

*Masking-Level Difference (MLD) Test [31, 32]*. In this dichotic test, binaural thresholds for a 500 Hz pure tone are determined in the presence of contralateral masking noise. The tone is either in-phase or out-of-phase between the subjects' two ears. Participants press a button when they detect the tone.

*Gap in Noise (GIN) Detection Test [33]*. In this test, subjects pressed a button when they detected a small gap (silent interval) imbedded within white noise. Most subjects cannot detect very brief (e.g., 2 msec) gaps, but most subjects can detect longer-duration gaps (10 msec or greater).

*Dichotic Digits Test [34]*. For this test, participants listened to four numbers presented to both ears. In each test item, two numbers were presented to one ear and two numbers were presented to the other ear. Participants repeated as many of the four digits as possible. This test has good sensitivity to central auditory system pathology while remaining relatively resistant to mild-to-moderate high-frequency sensorineural hearing loss [35].

*Spatial Release from Masking (SRM) Tests*. These tests assess the ability of listeners to make use of spatial and spectral cues in a test situation in which three speech streams are presented simultaneously [36]. Sentences are drawn from the Coordinate Response Measure (CRM) corpus [37] and each has the form “Ready [callsign] go to [color] [number] now.” For example, “Ready Charlie, go to red four now.” In the 0° condition, the target sentence and two competing sentences were played simultaneously from a source directly in front of the listener. In the 45° condition, the two competing sentences were played from sources 45° to the right and left of the 0° condition, while the source of the target sentence remained directly in front of the listener. Participants responded to these auditory stimuli via a computer touch screen.

### 2.6. Data Analysis

Mean and standard deviation values were calculated for each assessment and study group. Between-group comparisons were conducted using 2-tailed *t*-tests and applying appropriate Bonferroni corrections as needed. Pearson's correlation calculations were also made in certain instances as indicated in Results.

## 3. Results

Table 1 summarizes the characteristics of participants and results of their assessments.

### 3.1. Participant Characteristics

The PD group consisted of 35 adults (23 males and 12 females; mean age = 66.9 ± 11.2 years). The control group also consisted of 35 adults (31 male and 4 female participants; mean age = 65.4 ± 12.3 years) who had no history of PD or other neurological disorders.

For PD patients, the time since disease diagnosis averaged 7.9 ± 3.0 years. Evaluations of PD patients using the Hoehn and Yahr scale and the Schwab and England Activities of Daily Living Scale yielded the following: twenty PD patients were assessed at Hoehn and Yahr stage 1; six patients were at stage 2; and nine were at stage 3. The mean Hoehn and Yahr stage for the group of 35 PD patients was 1.7 ± 0.9. Three PD patients scored 100% on the Schwab and England scale; nineteen patients scored 90%; ten scored 80%; and three scored 70%. The mean Schwab and England score for the group of 35 PD patients was 86.2 ± 7.7%.

*Levodopa Use by PD Patients*. All PD patients except one used levodopa medication daily: he had not yet started using this medication. All of the patients attended 3 appointments for this study and were asked if they were “on” or “off” the effects of levodopa at each appointment. Of 105 total appointments, PD patients reported that they were “on” the effects of levodopa for 95 appointments, “off” for 7 appointments, and “in-between” for 3 appointments.

### 3.2. Questionnaire Data

*Hearing History Questionnaire*. This instrument includes questions such as, “Do you have difficulty hearing the words people are speaking?” Thirty-five PD patients gave the following responses to this question: “No,” 9; “Sometimes,” 16; and “Often,” 10. The control group gave the following responses to the same question: “No,” 10; “Sometimes,” 22; and “Often,” 3. To summarize, 74% of the PD patients and 71% of control group participants reported that they sometimes or often have difficulty in hearing words that people are speaking. Compared to the control group, a higher percentage of the PD group answered that they “often” have difficulty in hearing the words people are speaking (29% versus 9%). Seven PD patients used hearing aids, and 10 control group participants used hearing aids at the time of this assessment.

*Hearing Handicap Inventory for Adults (HHIA)*. PD patients had somewhat higher mean HHIA scores (18.6 ± 7.5 versus 14.1 ± 8.0) compared to control subjects (*p* < 0.03). A significantly greater percentage of PD patients (44%) than control subjects (25%) scored above 18 points on the HHIA, which indicates substantial perceived difficulty in hearing and communicating on a regular basis. A majority of subjects in both groups reported that they sometimes or often have difficulty in understanding callers on the telephone, hearing television or radio programs, or following conversations in noisy environments.

*Ratings of Daily Activity Abilities.* PD patients were asked to rate their abilities “during the past week” for 12 activities such as swallowing, handwriting, dressing, hygiene, falling, salivating, turning in bed, walking, and cutting food (these questions were taken from Part II of the Unified Parkinson's Disease Rating Scale [25]). For PD patients in this study, the total score on these 12 questions ranged from 3 to 27 (mean = 12.1 ± 5.0), with higher scores indicating greater difficulty on the collection of tasks. Total score on Ratings of Daily Activity Abilities was positively correlated with duration of PD (Pearson's *r* = 0.41; *p* = 0.014). These data, combined with Hoehn and Yahr and Schwab and England results, suggest that the majority of PD patients in this study were in the early, or less severe, stages of the disease.

### 3.3. Neuropsychological Test Results

*WRAT*. PD patients scored 62.8 ± 6.5 on this test, and control subjects scored 64.1 ± 7.3, indicating slightly above-average performance (based on age-corrected norms) for both groups [38]. There was no statistically significant difference in WRAT scores between the PD and control groups. These results might reflect the high percentage of subjects in this study who earned a bachelor's degree or higher level of education: 76% of PD patients and 61% of control subjects.

*RAVLT*. Mean RAVLT scores (total of trials 1 through 5) were 42.9 ± 7.8 for PD patients and 46.0 ± 11.5 for control subjects, which indicates normal performance for the age and education level of study participants [39]. The difference in mean scores between study groups was not statistically significant. Also, there was no statistically significant difference in the number of intrusions or repetitions made by the two study groups on this test.

*Beck Depression Inventory II (BDI-II)*. Mean BDI scores were 7.0 ± 4.8 for PD patients and 5.0 ± 7.0 for control subjects, indicating minimal or no depression for either group. The difference in mean scores between study groups was not statistically significant.

### 3.4. Pure Tone Audiometry

**Figure 1:**
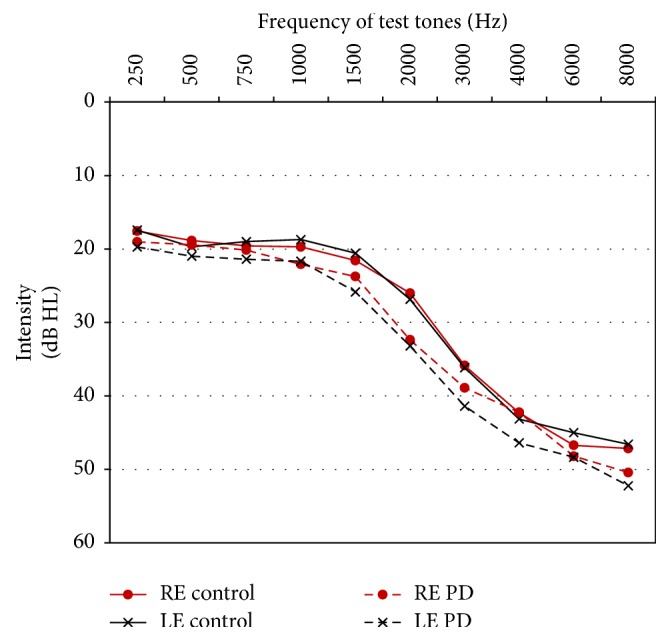
Mean pure tone air conduction thresholds for the PD and control groups. RE: right ear; LE: left ear.

Grand-averaged pure tone air conduction audiograms shown in Figure 1 indicate that both the control and PD groups had sloping, high-frequency sensorineural hearing loss which is typical for their age range. This degree of hearing loss would often interfere with a person's ability to hear certain environmental sounds and to understand speech, especially if background noise is present. Compared to control subjects, pure tone hearing sensitivity of PD patients was significantly worse for 1500 Hz (left ear, *p* = 0.012; right ear, *p* = 0.033) and 2000 Hz (left ear, *p* = 0.008; right ear, *p* = 0.024) test frequencies in both ears. Pure tone average (PTA) thresholds for frequencies of 0.5, 1, 2, and 4 kHz in the worse ear were 28.9 ± 14.1 dB HL for the control group and 33.9 ± 13.0 dB HL for the PD patients, indicating mild-to-moderate hearing loss for both groups, which was slightly worse for PD patients. The difference in PTA thresholds between groups was not statistically significant (*p* = 0.13). Also, there were no statistically significant within-group differences between the left and right ears for any of the test frequencies.

Based on their audiometric results, 27 PD patients (77%) were good candidates for (that is, they would probably benefit from) hearing aids. Of these 27 hearing aid candidates, only 7 (26%) owned and used the devices. A smaller percentage of control subjects (54%) were good candidates for hearing aids. Of these, 54% used hearing aids. Worse audiometric thresholds for 1500 and 2000 Hz tones exhibited by PD patients (compared to control subjects) contributed to the greater percentage of them being candidates for hearing aids.

### 3.5. Assessments of Central Auditory Processing

*Speech intelligibility in noise *was assessed using the Words in Noise (WIN) test. As a group, control subjects scored a mean of 19.4 ± 7.6 items correct on this test, and PD patients scored 18.2 ± 5.9 items correct for the right ear. Left ear scores were 19.1 ± 6.3 for the control group and 18.1 ± 6.4 for the Parkinson group. Although group differences did not reach statistical significance (possibly due to intersubject variability), these results indicate that both groups have impaired ability to understand speech in noisy environments, a common complaint of older people who have significant hearing loss. The association between the degree of hearing loss and difficulty in understanding speech in noise is demonstrated by the significant negative correlation between PTA and WIN score for the control group (*r* = −0.83 for the left ear and −0.75 for the right ear) and the PD group (*r* = −0.74 for the left ear and −0.75 for the right ear) (see Table 2).

*Staggered-Spondaic-Word (SSW) Test*. PD patients made a mean of 14.9 ± 12.5 total errors on this test, while control subjects averaged 14.2 ± 16.9 errors. The difference in mean scores between study groups was not statistically significant. These results indicate that both groups exhibit central auditory and speech processing deficits (again, a consequence of their age and hearing loss). By comparison, a group of 29 younger (mean age = 32 years) healthy control subjects with normal hearing from a study by Gallun et al. [40] averaged only 4.1 ± 3.0 total errors on the SSW test.

*Masking-Level Difference (MLD) Test*. Mean scores on this test were 10.8 ± 2.2 dB for PD patients and 10.1 ± 3.7 dB for the control group, indicating no statistically significant difference between these groups. For this test, a higher MLD value signifies better performance. A group of 29 younger (mean age = 32 years) healthy control subjects from Gallun et al.'s study [40] with normal hearing averaged 13.6 ± 2.8 dB for this test, which is significantly better performance than either of the older groups in the current study.

*Gap in Noise (GIN) Detection Test*. The mean gap detection threshold for both the control and PD patient groups was 8.9 msec for the right ear and 9.6 msec for the left ear. By comparison, the mean gap detection threshold for the group of 29 young control subjects in Gallun et al.'s study [40] was 3.8 msec for the right ear and 4.3 msec for the left ear. Because the gap in noise detection test is designed to simulate subjects' ability to distinguish gaps within speech, poorer performance by PD patients and older control subjects in this study indicates impaired central auditory processing that contributes to their difficulties in understanding speech in noisy environments.

*Dichotic Digits Test*. There was no statistically significant difference in performance between the PD and control groups on this test for either left or right ear stimuli. For right ear stimuli, the PD group's mean score was 92.7 ± 6.9% correct, while the control group scored 88.2 ± 12.3% correct. For left ear stimuli, the PD group's mean score was 85.6 ± 13.8% correct, while the control group scored 84.3 ± 13.2% correct.

*Spatial Release from Masking (SRM) Tests*. PD patients scored worse (mean = 6.6 ± 1.7) than control subjects (mean = 7.5 ± 2.5) when target sentences and competing sentences were all presented at 0°, although this difference did not quite reach statistical significance (*p* < 0.08). The PD group also scored worse (mean = 8.3 ± 3.0) than control subjects (mean = 11.2 ± 3.9) when target sentences and competing sentences were separated in space by 45° (*p* < 0.002). These results indicate that, compared to the control group, the PD group had greater difficulty in understanding sentences in a background of competing speech, and they also showed less improvement on this task when the target and competing sentences were separated in space.

### 3.6. Correlations among Assessments and Other Variables

Table 2 contains statistically significant Pearson's correlation values for pertinent variables and assessments. Participants' age was significantly correlated with the pure tone average (PTA) air conduction threshold for both the PD (*p* = 0.02) and control (*p* = 0.0003) groups. Age was also correlated with RAVLT total score in the PD group (*p* < 0.0001) but not in the control group (*p* = 0.39). Significant correlations were found between age and several central auditory assessments (WIN test in the left ear, GIN detection test in the left ear, Dichotic Digits Test in the left ear, and spatial release from masking test for the 45° condition) for the control group. However, the PD group only exhibited a significant correlation between age and WIN test in the left ear (*p* = 0.02). In all of these examples, greater age was associated with poorer performance on assessments.

In addition to age, pure tone average (PTA) air conduction threshold also correlated significantly with several assessments: WIN test (both ears), Dichotic Digits Test in the right ear, and HHIA score for both groups; GIN detection test in the right ear, SSW test, and spatial release from masking test (for the 45° condition) for the control group; and RAVLT score for the PD group (*p* = 0.01). In all of these examples, greater PTA air conduction threshold was associated with poorer performance on assessments.

## 4. Discussion

### 4.1. Questionnaire Data

Compared to the control group, PD patients in this study reported greater difficulty in hearing words people are speaking. Also, PD group's scores on the HHIA questionnaire were significantly higher than control group's scores. These results demonstrate that many PD patients recognize and acknowledge their hearing loss and resultant problems with communication. This underscores the need for early diagnosis and remediation of these conditions within the PD population.

### 4.2. Neuropsychological Tests

Results of the two neuropsychological tests used in this study (WRAT and RAVLT) did not reveal significant cognitive decline for either group or significant differences in performance between the PD and control groups. Reasons for these results might include the following:

1. There is insufficient sensitivity of the tests used (WRAT and RAVLT) for this population.
2. The relatively high education levels achieved by both groups, especially the PD group, might provide some degree of cognitive compensation/protection against the effects of aging or PD.
3. Most of the PD patients in this study were in the early or less severe stages of the disease. It is likely that many of these patients will experience cognitive decline as they age and their disease progresses. Unfortunately, some PD patients will also experience increases in depression for the same reasons.

### 4.3. Pure Tone Audiometry

Compared to age-matched healthy control subjects, PD patients exhibited worse hearing sensitivity for 1500 and 2000 Hz test frequencies. Audiometric results from this study are different from those published by Yýlmaz et al. [14] who reported that a group of 20 PD patients had worse hearing at 4000 and 8000 Hz compared to a group of age-matched control subjects. The most likely reason for this disparity in findings is the relatively small number of subjects in each of these studies. In order to draw definitive conclusions regarding differences in audiometric results between PD patients and age-matched control subjects, it would be necessary to collect and analyze data from much larger pools of participants (as, e.g., in [3] or [4]). However, worse thresholds for 1500 and 2000 Hz tones exhibited by PD patients in the current study reflect a pattern of hearing loss which is more likely to be noticed by patients compared to similar degrees of hearing loss at 4000 or 8000 Hz. In this study, PD patients and control subjects both exhibited a pattern of high-frequency hearing loss which is typical for their age [3, 4]. These results are similar to those reported by Vitale et al. [15] in a study of 106 PD patients. In that study, the pure tone average (PTA) threshold for audiometric frequencies of 0.5, 1, 2, and 4 kHz was 26 dB for the entire patient group, with greater degrees of hearing loss exhibited by older subgroups of participants.

Although sensorineural hearing loss cannot be “cured,” effective rehabilitative strategies exist, which can ameliorate many of adverse effects of HL, which include communication difficulties, social withdrawal, isolation, fatigue, frustration, depression, cognitive decline, and dementia [11, 41–47]. Numerous studies have demonstrated that implementation of auditory rehabilitation strategies contributes to improvements in communication, cognitive functioning, and quality of life for people with significant hearing loss [48–52].

It is especially important for PD patients to optimize communication with their family members, caregivers, friends, and clinicians, including pharmacists. Also, it is important for PD patients who develop hypophonia and other problems with speech production to be able to hear themselves and feedback from others (including speech pathologists) as clearly as possible [53, 54]. In a study of elderly patients without PD, Cohen and Turley [55] reported that subjects with hearing loss were more likely to have dysphonia than those without hearing loss. Subjects with both dysphonia and hearing loss had greater depression scores than those with neither symptom. Cohen and Turley concluded that “voice problems and hearing loss are common in the elderly, adversely impact quality of life, and require simultaneous management.” These statements certainly apply to PD patients. In fact, De Keyser et al. [56] concluded, “Auditory perceptual deficits may influence speech production in patients with PD.”

Another reason to assess and remediate hearing loss experienced by PD patients is the fact that auditory cues are sometimes used during training protocols to improve gait and other sequential movements in this population [57–62]. PD patients with significant, untreated hearing loss would have difficulty in perceiving and differentiating between auditory cues.

### 4.4. Assessments of Central Auditory Processing

Both of the study groups exhibited significant deficits in many assessments of central auditory processing (CAP), with PD patients performing worse than the control group on the spatial release from masking (SRM) test. It is likely that as they age and their disease progresses, PD patients will exhibit more severe CAP deficits over time. These deficits can contribute to communication problems, including the ability to hear speech clearly and to extract meaning from spoken language. Auditory processing disorders can also impair an individual's ability to detect and understand speech in noisy conditions and to locate the source of sounds. In addition to other symptoms endured by PD patients, these auditory deficits can contribute to decreased quality of life, social isolation, frustration, and depression.

A few other studies assessed central auditory processing by PD patients. For example, Guehl et al. [18] reported gap detection thresholds of approximately 5 msec for a group of 19 PD patients, which were significantly shorter than the GIN detection thresholds exhibited by PD patients in the current study (9 msec). However, Guehl et al.'s PD patients were 10 years younger (on average) and had significantly better audiometric thresholds compared to PD patients in the current study.

Lewald et al. [19] investigated auditory spatial perception in a PD population by employing a simple task involving left/right judgments about dichotic stimuli presented with various interaural time differences (ITD). The acuity of sound lateralization was significantly reduced in PD: the just noticeable difference (JND) in interaural time recorded for PD patients was about twice that observed for age-matched healthy controls. Lewald et al. postulated that this deficit may be related to a potential role of the basal ganglia in spatial hearing functions.

Vitale et al. [20] compared speech reception thresholds (SRT) and word recognition scores (WRS) of 45 PD patients with those from 45 age-matched healthy control subjects. While both groups exhibited similar levels of high-frequency sensorineural hearing loss, mean values for the SRT were higher in PD patients (right ear: PD, 37.0 ± 12.9, and controls, 29.9 ± 13.22; left ear: PD, 39.2 ± 14.14, and controls, 29.3 ± 16.9). Also, WRS results indicated that only 49% of the PD group exhibited normal speech perception profiles, compared to 78% of the control group.

Results of the current study and those reported by Guehl et al. [18], Lewald et al. [19], and Vitale et al. [20] indicate impaired neural processing of auditory stimuli by PD patients. Several factors probably contribute to these central auditory processing (CAP) deficits, including aging, hearing loss, and degeneration/dysfunction of neural structures and pathways related to the pathophysiology of PD [15]. While these factors cannot yet be stopped or reversed, their negative effects on central auditory processing might be minimized or slowed by implementing effective and appropriate aural rehabilitation strategies, which may include the following:

1. Amplification via hearing aids, cochlear implants, or other devices. In this study, only 26% of PD patients who would benefit from amplification used hearing aids. Appropriate amplification can improve hearing ability, speech understanding, and sound localization and might also help to reduce the patient's risk or severity of cognitive decline, anxiety, and depression [49, 50, 63–66]. Davis et al. [67] stated that hearing aid candidates who were identified early had greater benefit through additional years of hearing aid use and better adaptation to use compared to those of the same age and hearing impairment who were fitted with hearing aids later
2. Assistive listening devices including amplified telephones, TV listening devices, and personal FM systems for use in public settings such as lectures, plays, or religious services. Also, visual alerting devices can increase awareness of alarms and doorbell rings
3. Communication optimization strategies, which include good environmental lighting, decreasing background noise, and encouraging speakers to do the following:
  - Speak at a reasonable rate
  - Speak when their face can be seen clearly (keep their hands away from their face)
  - Get the listener's attention before speaking
  - Speak to people from a reasonable distance (3–6 ft), not from a different room

Additional information on aural rehabilitation may be obtained from licensed audiologists and from the American Speech-Language-Hearing Association's web site: http://www.asha.org/public/hearing/Adult-Aural-Rehabilitation.

## 5. Limitations of This Study

Because the sample size of this study was relatively small, our conclusions regarding auditory or cognitive deficits associated with PD should be interpreted in context. An extensive battery of cognitive assessments was not included in the study design; therefore, we collected limited data on cognitive function of participants aside from auditory processing. Also, most of the PD patients who participated in this study were in the early or less severe stages of the disease. Therefore, we do not know how more severe PD might affect auditory processing. Finally, because there was a majority of males in our study sample, especially in the control group, we cannot make any assumptions about the performance of males versus females in this population.

## 6. Conclusions

Because of the many physical, emotional, and cognitive challenges that PD patients will face as their disease progresses, it is vital to identify and remediate auditory dysfunction in this population as early as possible. It is imperative to implement rehabilitative strategies that will improve PD patients' ability to hear and communicate. After these strategies are implemented, increased quality and enjoyment of life for PD patients should result from (a) improved ability to communicate with family members, friends, clinicians, and other people, (b) enhanced ability to hear music and environmental sounds, and (c) improved comprehension of telephone conversations, television and radio programs, religious services, and theater productions.

## Figures and Tables

**Table 1:** Characteristics of participants and results of assessments.

	PD group (*n* = 35)	Control group (*n* = 35)	Between-group comparison
Age (years), mean ± SD	66.9 ± 11.2	65.4 ± 12.3	NS
Duration of PD (years), mean ± SD	7.9 ± 3.0	N/A	N/A
Wide Range Achievement Test (WRAT), mean ± SD	62.8 ± 6.5	64.1 ± 7.3	NS
Rey Auditory Verbal Learning Test (RAVLT) trials 1–5 (mean ± SD)	42.9 ± 7.8	46.0 ± 11.5	NS
Beck Depression Inventory (BDI) score (mean ± SD)	7.0 ± 4.8	5.0 ± 7.0	NS
Hearing Handicap Inventory score (mean ± SD)	18.6 ± 7.5	14.1 ± 8.0	*p* < 0.03
Pure tone average air conduction hearing threshold (dB HL) in the worst ear ± SD	33.9 ± 13.0	28.9 ± 14.1	*p* = 0.13
Words in Noise (WIN) test score, right ear (mean ± SD)	18.2 ± 5.9	19.4 ± 7.6	NS
Words in Noise (WIN) test score, left ear (mean ± SD)	18.1 ± 6.4	19.1 ± 6.3	NS
Staggered-Spondaic-Word (SSW) test, total errors (mean ± SD)	14.9 ± 12.5	14.2 ± 16.9	NS
Masking-Level Difference (MLD) test score (dB), mean ± SD	10.8 ± 2.2	10.1 ± 3.7	NS
Gap in Noise (GIN) detection (msec), mean	8.9 (right ear)	8.9 (right ear)	NS
	9.6 (left ear)	9.6 (left ear)	NS
Dichotic Digits Test score (% correct), mean ± SD	92.7 ± 6.9 (right)	88.2 ± 12.3 (right)	NS
	85.6 ± 13.8 (left)	84.3 ± 13.2 (left)	NS
Spatial release from masking (SRM) test score, 0° condition (mean ± SD)	6.6 ± 1.7	7.5 ± 2.5	*p* < 0.08
Spatial release from masking (SRM) test score, 45° condition (mean ± SD)	8.3 ± 3.0	11.2 ± 3.9	*p* < 0.002

NS, not statistically significant.

**Table 2:** Pearson's correlation (*r*) values for pertinent variables and assessments.

Factor	Covariate	PD group (*n* = 35)		Control group (*n* = 35)	
		Pearson's *r*	*p*	Pearson's *r*	*p*
Participants' age	Pure tone average air conduction hearing threshold (dB HL) in the worst ear	0.383	0.02	0.565	0.0003
	Words in Noise (WIN) test score, right ear	−0.209	0.22	−0.276	0.10
	Words in Noise (WIN) test score, left ear	−0.377	0.02	−0.375	0.02
	Gap in Noise (GIN) detection, left ear	0.275	0.10	0.481	0.003
	Dichotic Digits Test (DDT), left ear	0.082	0.63	0.416	0.01
	Spatial release from masking test, 45° condition	−0.115	0.50	−0.441	0.007
	RAVLT (total of trials 1 through 5)	−0.647	0.0001	−0.148	0.39

Pure tone average (PTA) air conduction hearing threshold (dB HL) in the worst ear	Words in Noise (WIN) test score, right ear	−0.750	0.0001	−0.754	0.0001
	Words in Noise (WIN) test score, left ear	−0.741	0.0001	−0.827	0.0001
	Staggered-Spondaic-Word test	0.312	0.06	0.669	0.0001
	Gap in Noise (GIN) detection, right ear	0.201	0.24	0.570	0.0003
	Dichotic Digits Test (DDT), right ear	0.363	0.03	0.512	0.001
	Spatial release from masking test, 45° condition	−0.121	0.48	−0.560	0.0004
	RAVLT (total of trials 1 through 5)	−0.420	0.01	−0.242	0.16
	Hearing Handicap Inventory score	0.323	0.05	0.668	0.0001
